# CD44v6 Regulates Growth of Brain Tumor Stem Cells Partially through the AKT-Mediated Pathway

**DOI:** 10.1371/journal.pone.0024217

**Published:** 2011-09-06

**Authors:** Mayumi Jijiwa, Habibe Demir, Snehalata Gupta, Crystal Leung, Kaushal Joshi, Nicholas Orozco, Tiffany Huang, Vedat O. Yildiz, Ichiyo Shibahara, Jason A. de Jesus, William H. Yong, Paul S. Mischel, Soledad Fernandez, Harley I. Kornblum, Ichiro Nakano

**Affiliations:** 1 Department of Neurological Surgery, The Ohio State University, Columbus, Ohio, United States of America; 2 Jonsson Comprehensive Cancer Center, David Geffen School of Medicine, University of California Los Angeles, Los Angeles, California, United States of America; 3 Department of Molecular and Medical Pharmacology, David Geffen School of Medicine, University of California Los Angeles, Los Angeles, California, United States of America; 4 Department of Pathology, David Geffen School of Medicine, University of California Los Angeles, Los Angeles, California, United States of America; 5 Center for Biostatistics, The Ohio State University, Columbus, Ohio, United States of America; 6 James Comprehensive Cancer Center, The Ohio State University, Columbus, Ohio, United States of America; The University of Chicago, United States of America

## Abstract

Identification of stem cell-like brain tumor cells (brain tumor stem-like cells; BTSC) has gained substantial attention by scientists and physicians. However, the mechanism of tumor initiation and proliferation is still poorly understood. CD44 is a cell surface protein linked to tumorigenesis in various cancers. In particular, one of its variant isoforms, CD44v6, is associated with several cancer types. To date its expression and function in BTSC is yet to be identified. Here, we demonstrate the presence and function of the variant form 6 of CD44 (CD44v6) in BTSC of a subset of glioblastoma multiforme (GBM). Patients with CD44^high^ GBM exhibited significantly poorer prognoses. Among various variant forms, CD44v6 was the only isoform that was detected in BTSC and its knockdown inhibited *in vitro* growth of BTSC from CD44^high^ GBM but not from CD44^low^ GBM. In contrast, this siRNA-mediated growth inhibition was not apparent in the matched GBM sample that does not possess stem-like properties. Stimulation with a CD44v6 ligand, osteopontin (OPN), increased expression of phosphorylated AKT in CD44^high^ GBM, but not in CD44^low^ GBM. Lastly, in a mouse spontaneous intracranial tumor model, CD44v6 was abundantly expressed by tumor precursors, in contrast to no detectable CD44v6 expression in normal neural precursors. Furthermore, overexpression of mouse CD44v6 or OPN, but not its dominant negative form, resulted in enhanced growth of the mouse tumor stem-like cells *in vitro*. Collectively, these data indicate that a subset of GBM expresses high CD44 in BTSC, and its growth may depend on CD44v6/AKTpathway.

## Introduction

Glioblastoma multiforme (GBM) is one of the most lethal types of human cancers, with a median patient survival of 12–15 months [1], [2]. Current therapy, including surgery followed by chemotherapy and radiation, is generally only palliative and does not result in marked improvement in patient survival [2], [3]. Although the initial treatment regimen generally shrinks the tumor mass, recurrence of the tumor is virtually inevitable, suggesting that at least a subset of tumor cells is resistant to therapy [2]. Emerging evidence indicates that at least some of this resistance is mediated by brain tumor stem-like cells (BTSC) [4], [5]. Therefore, identification of BTSC inhibitors is a high priority for the development of effective GBM therapies. However, the development of therapies directed against BTSC is complicated partly due to the fact that they are heterogeneous, lacking a definitive marker set, even within tumors of the same histopathological types [6], [7], [8].

CD44 is a cell surface protein expressed in multiple types of tumors. It is also expressed in certain normal tissues where it functions in the regulation of cell proliferation, cell migration, transmission of survival signals, and other cell-cell and cell-matrix interactions [9], [10], [11], [12], [13] demonstrated that CD44 antagonists attenuate *in vivo* growth of mouse tumors derived from glioma cell lines, suggesting that CD44 is a potential therapeutic target for GBM. Further, Anido et al. [14] recently reported that GBM tumor initiation is attenuated by targeting TGF-b and its receptor CD44 *in vivo*.

Recent studies, however, have been inconclusive regarding which isoforms of CD44 are the key molecules in BTSC. CD44 exists as a large family of isoforms, produced by the alternative splicing of up to 20 exons, which generate different binding sites for the molecule [9], [15]. Exons 1–5 and 16–19 are spliced together to form the transcript for CD44s (s for standard form), which is expressed in a wide range of normal tissues as well as in tumors of ectodermal origin [15]. Exons 6–15 are alternatively spliced into the mRNA to form the variable exons v1–v10 [11], [15], [ and 16]. These variant isoforms are expressed in many different organs and have been strongly linked to tumor progression behaviors in various cancers [15], [17], [18], [19]. The variant 6 isoform (CD44v6), in particular, is associated with several cancer types but not in somatic cells [17], [20], [21]. CD44v6 and its ligand, osteopontin (OPN) are highly expressed in breast cancer [20], [22] as well as leukemia [23] and gastric cancer [24], and regulate malignant transformation by inducing tumor cell proliferation and controlling migration [20], [21].

Expression of CD44 is widely identified in cancer stem cells in various organs, such as breast, colon, and pancreas [25], [26], [27]. In these tumors, tumor-forming cells *in vivo* are restricted to CD44-positive tumor cells [25], [27]. However, little is known about which isoforms are specifically associated with cancer stem cells. A recent study demonstrated that CD44v6 is likely expressed by bladder carcinoma stem cells, suggesting that this isoform may be of particular link to cancer stem cells [28]. To our knowledge, no study has identified the specific CD44 isoforms that are present in BTSC.

Here, we demonstrate that a subset of BTSC in GBM express CD44 and its variant form 6 (CD44v6) plays a positive role in their growth *in vitro*. CD44-neutralizing antibody inhibits the growth of BTSC derived from GBM samples that contain cells with high CD44 (CD44^high^ GBM), while it has no significant effect on the BTSC from GBM containing population of cells with low expression of CD44 (CD44^low^ GBM). BTSC derived from CD44^high^ GBM depend on CD44v6 to maintain proliferation. Targeting CD44v6 has, therefore, potential impact in eradicating therapy-resistant BTSC in GBM.

## Materials and Methods

### Ethics

Experiments using human tissue-derived materials were carried out under the approved institutional review board at UCLA. Informed consent was obtained in written form from all human subjects and families of autopsy patients to publication of their case details prior to the experiments performed in this study. All animal experimentation was performed with the approval of the UCLA Animal Research Committee, following NIH guidelines, using animal protocol number 93-285. The mice were experimentally used in accordance with the Institutional Animal Care and Use Committee guidelines at UCLA.

### Tissue culture

Tumors derived from mice deficient in both p53 and patched were kindly provided by Dr. James A. Waschek at UCLA [29]. Neurosphere (NS) cultures were prepared as previously described [30]. Briefly, small GBM samples were dissociated with a fire-polished glass pipette and resuspended at 50,000 cells/ml in neurosphere medium, containing Dulbecco's modified Eagle medium (DMEM)/F12 medium (GIBCO, Invitrogen, Carlsbad, CA) supplemented with B27 (final concentration 2%, GIBCO, Invitrogen, Carlsbad, CA), basic fibroblast growth factor (bFGF) (20 ng/ml, Peprotech, Rocky Hill, NJ), epidermal growth factor (EGF) (50 ng/ml, Peprotech), penicillin/streptomycin (1%, GIBCO, Invitrogen, Carlsbad, CA), and heparin (5 µg/ml, Sigma Aldrich, St. Louis, MO). To differentiate the BTSC, spheres were dissociated into single cells, added to poly-L-Lysine coated dishes containing Neurobasal medium (GIBCO, Invitrogen, Carlsbad, CA) with B27, and maintained for up to five days, followed by culture in serum containing medium.

### RT-PCR

Total RNA was isolated with TRIzol (GIBCO, North Andover, MA) from GBM specimens and adjacent normal brains of autopsy samples. One µg of each total RNA was reverse transcripted to cDNA with ImProm-II Reverse Transcriptase (Invitrogen for conventional polymerase chain reaction (PCR), Promega, Madison, WI, for quantitative PCR), according to the manufacturer's protocol. Reverse transcription-polymerase chain reaction (RT-PCR) with primers for the glyceraldehyde-3-phosphate-dehydrogenase (GAPDH) gene served as an internal control. After correcting for GAPDH signals by electrophoresis, reverse transcripted cDNA was subjected to quantitative PCR analysis using the gene specific primers. The primers for the CD44; forward: TTTGCATTGCAGTCAACAGTC and reverse: GTTACACCCCAATCTTCATGTCCAC, for the CD44v6; forward: GAAGAAACAGCTACCCAGAAGGAACAG and reverse: GCCAAGAGGGATGCCAAGATG and for the GAPDH; forward: AAGGTGAAGGTCGGAGTCAA and reverse: AATGAAGGGGTCATTGATGG were constructed based upon GenBank accession number NM000610. The protocol for the thermal cycler was described previously [30]. Control experiments excluded reverse transcriptase and/or template cDNA. Each reaction was visualized after electrophoresis with 2% agarose gel. Relative quantification for quantitative real-time polymerase chain reaction (qRT-PCR) was determined with the LightCycler Relative Quantification Software (Roche Diagnostics, Indianapolis, IN).

### Western blot

Total lysate of brain tissue were prepared from GBM specimens and adjacent normal brains of autopsy samples using sodium dodecyl sulfate (SDS) sample buffer. Whole-cell lysates were prepared in lysis buffer containing Protease Inhibitor CockTail (P8340, Sigma Aldrich, St. Louis, MO) and protein concentrations determined by bicinconic acid (BCA) protein assay kit (Thermo SCIENTIFIC, Rockford, IL) according to the manufacturer's protocol. Equal amounts of proteins were fractionated on sodium dodecyl sulfate-polyacrylamide gel electrophoresis and transferred to polyvinylidene fluoride (PVDF) membrane (Invitrogen, Carlsbad, CA). The membrane was incubated with AKT (rabbit, 1∶1000, Cell Signaling Technology, Danvers, MA), phospho-AKT (rabbit, 1∶1000, Cell Signaling Technology, Danvers, MA), and GAPDH antibody (rabbit, 14C10, Cell Signaling Technology), followed by signal amplification with anti-rabbit immunoglobulin G (1∶250, GE Healthcare, Pataskala, OH) and detection with enhanced chemiluminescence.

### Tissue Microarray

Tissue microarray (TMA) consisting of three to six representative 0.6-mm cores from formalin-fixed, paraffin-embedded tissue blocks was generated at the Department of Pathology and Laboratory Medicine at UCLA, under the protocols approved by the UCLA Institutional Review Board. The tissue samples were collected either from autopsies of patients with GBM within 24 hours of death or from patients who underwent surgery at UCLA Medical Center. After immunohistochemistry, tissues too small and/or crushed were eliminated, and 64 samples from 37 patients were introduced to further analysis. All samples were diagnosed as high grade glioma (corresponding to glioblastoma and anaplastic astrocytoma) or low grade glioma by nuclear atypia and cell density, or tumor-free region. CD44 expression was analyzed by two neuropathologists in a blind protocol, and staining patterns were determined according to the immunoreactive site as cell surface and process. Overall staining intensity was scored as − (negative), + (weak), ++ (moderate to strong). For the characterization of TMA samples and appropriate patients, highest grade tissues of patients showing multiple features were adopted. Overall survival period was defined as from initial diagnosis to decease.

### Immunohistochemistry

Surgical GBM tissues were fixed in 10% formaldehyde and embedded in paraffin. Slides were deparaffinized in xylene and rehydrated with ethanol. For the antigen retrieval, slides were immersed in 0.01 M citrate buffer (Thermoscientific Fisher, Rockford, IL), pH 6, and heated with a microwave for 10 minutes. Peroxidase activity was quenched with 0.3% hydrogen peroxide in methanol. After blocking for one hour with 10% normal goat serum, slides were incubated with mouse anti-human monoclonal CD44 antibody, Phagocytic Glycoprotein-1 (Clone DF1485, Dako, Carpentaria, CA) for two hours at room temperature. Antibody binding was detected using EnVision+ (Goat, anti-mouse,Dako), followed by Vector DAB substrate kit (Vector Laboratories, Burlingame, CA), counterstained with Hematoxylin.

### Immunocytochemistry

Immunocytochemistry of GBM neurospheres using the CD44 antibody (BioLegend, San Diego, CA) was performed as previously described [30]. Primary antibody was visualized with Alexa 488 (Cell Signaling Technology, Danvers, MA), and Hoechst 333342 (Sigma Sigma Aldrich, St. Louis, MO) was used for a fluorescent nuclear counterstain.

### Flow cytometry

Cells were incubated with CD44 or CD44v6 antibody (BioLegend, San Diego, CA), or CD133 antibody (1∶200, Miltenyi Biotec, Bergisch Gladbach, Germany), conjugated to Alexa 488 (Cell Signaling Technology, Danvers, MA) for 30 minutes at room temperature and separated into positive and negative fractions using fluorescence-activated cell sorter, FACSCalibur (Becton, Dickinson and Company, Franklin Lakes, NJ). Gating parameters were set by side and forward scatter to eliminate dead and aggregated cells. Apoptosis assay was performed using the Apoptosis Detection Kit (R&D Systems, Minneapolis, MN), according to the manufacturer's instructions. U87 glioma cell line was purchased from American Type Culture Collection (Rockville, MD).

### Sphere forming assay

To assay for sphere-forming potential, 100 cells from dissociated GBM neurospheres were plated on each well of 96-well plates with NS media, and the number of neurospheres were counted at day seven [30]. The small interfering RNA (siRNA) transfectants were removed from plates with TrypLE Express (GIBCO, Invitrogen, Carlsbad, CA) and replated to 96-well plates six hours after transfection.

### Xenotransplantation of tumor spheres into NOD/SCID mice

Nonobese diabetic/severe combined immunodeficiency (NOD/SCID) mice of 6–8 weeks of age (Charles River Laboratories, Wilmington, MA) were anesthetized with intraperitoneal administration of ketamine. GBM neurospheres were dissociated and 500,000 cells were stereotactically transplanted in the right striatum. After 12 weeks, the mice were undergone intracardiac perfusion-fixation with 4% paraformaldehyde. Brains were removed and retrieved for frozen sections, followed by hematoxylin and eosin staining, except for the tumor derived from GFP expressing cells.

### siRNA construction and transfection

siRNA was synthesized using the Silencer siRNA Construction Kit (Applied Biosystems/Ambion, Austin, TX), according to the manufacturer's protocol. The two sequences targeting CD44v6 are: sense1. AATTGTACTACTAGGAGTTGCCCTGTCTC; antisense1. GCAACTCCTAGTAGTAC AATTCCTGTCTC; sense2. AATGTTT GGCGATATCCCTCACCTGTCTC; antisense2. TGAGGGATATCGCCAAACATTCCTGTCTC. Transfection using Lipofectamine2000 (Invitrogen, Carlsbad, CA) was performed as previously described [30].

### PI3K/AKT signal inhibition

Stock solutions of inhibitors for AKT (AKT V/IX/X), PI3K (LYS294002), and rapamycin (Calbiochem, San Diego, CA) were made by dissolving in dimethyl sulfoxide (DMSO) (Sigma Aldrich, St. Louis, MO) and stored at −20°C. Inhibitors were added to each well at final concentrations of 1, 3, and 10 µM, respectively. For combined treatment with siRNA transfeciton, 1 µM of AKT inhibitor X was used. An equal concentration of DMSO served as control.

### Statistical analysis

Results were analyzed by using SAS version 9.2 and STATA 10. For parametric data; Statistical Analysis was performed by using 1-way ANOVA and t-test followed by Bonferroni post hoc testing and Repeated measurement ANOVA followed by Tukey post hoc testing. For nonparametric data; Wilcoxon Rank test was used followed by Bonferroni post hoc testing. Additionally for the count data Fisher Exact test was used to investigate the relationship between two categorical data. Significance was accepted if p<0.05.

## Results

### Overall CD44 expression is elevated in GBM

**Figure 1:**
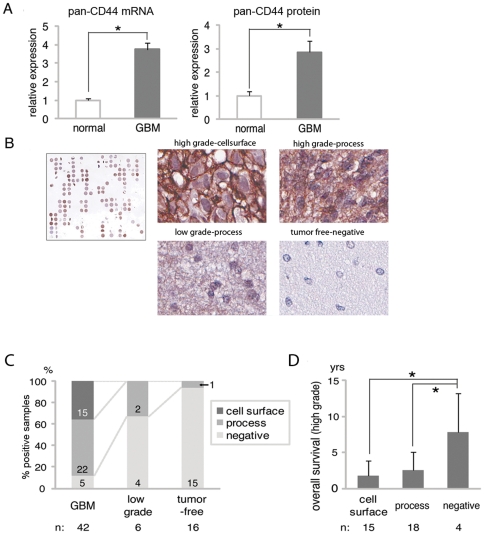
Expression of CD44 in glioma and clinical prognosis. A: The average level of mRNA (left panel) and protein (right panel) of CD44 were upregulated in GBM. Samples from five normal and 20 GBM cases were analyzed. B: Immunohistochemistry of 64 specimens from 37 GBM patients (left panel). Representative staining pattern showing diagnostic criteria (middle and right panels). Magnification ×60. C: The analysis of different grades of gliomas and tumor free regions, and their correlation with the localization of CD44 expression. The number in each column indicates the number of appropriate samples. D: The analysis of patient survival in high grade gliomas with respect to localization of CD44 expression. “n” indicates the number of appropriate patients. Graph showing the number of cases with negative (−), positive (+), or highly positive (++) immunoreactivity for CD44 on cell surface and process of either primary specimens (gray) or recurrent specimens (black) (right panel). *, p<0.05. For figure 1C, Fisher Exact test was performed to analyze the data. For Figure 1D, Log transformation was applied and one way ANOVA method was performed to analyze the data. Results represented as means ± S.D.

To investigate the roles of the CD44 pathway in GBM, we first examined the expression of pan-CD44 (all CD44 isoforms) in GBM surgical specimens. Quantitative RT-PCR demonstrated that the average of pan-CD44 mRNA expression from 20 cases of GBM was higher than that from five adjacent normal brains (Fig. 1A, left panel). Likewise, the average of pan-CD44 protein expression in GBM, determined by Western blot, was higher than that of adjacent normal brains (Fig. 1A, right panel).

### Expression of pan-CD44 is associated with poorer prognosis in GBM

Next, we investigated the potential link between CD44 expression and patient prognosis. Tissue microarray containing 64 tumor samples from 37 patients was performed to assess any correlation between pan-CD44 protein expression and the overall survival of the affected patients (Fig. 1B, 1C and 1D). Patients included in this analysis were initially diagnosed as low grade astrocytoma (n = 3), anaplastic astrocytoma (n = 5), or GBM (n = 29), and all patients eventually developed GBM (Table 1). In accordance with previous findings [31], [32] we analyzed the immunohistochemical staining results with respect to two different features: histopathological grade and the subcellular localization of pan-CD44. The evaluated samples contained 42 GBMs, 6 low grade gliomas, and 16 tumor-free regions. Figure 1B shows representative pan-CD44 staining patterns of GBM (Fig. 1B, upper middle and upper right panels), low grade glioma (Fig. 1B, lower middle panel), and tumor-free region (Fig. 1B, lower right panel). Subcellular localization of pan-CD44 immunoreactivity was divided into 3 patterns; cell surface (Fig. 1B, upper middle panel), process (Fig. 1B, upper right and lower middle panels), and negative (Fig. 1B, lower right panel). Pan-CD44 immunoreactivity on cell surface was identified only in GBMs, whereas low grade gliomas and tumor-free regions exhibited faint or no signal on the processes (Fig. 1C). Furthermore, among 37 patients, the average of overall survival of pan-CD44-positive cases was significantly shorter than the pan-CD44-negative cases (Fig. 1D). This also agrees with a recent study by Anido et al. [14]. These data suggest that pan-CD44 immunoreactivity is linked to pathological malignancy as well as poorer prognosis of patients with GBM.

**Table 1:** Characteristics of GBM samples used for the first TMA analysis.

1. Characteristics of GBM samples for TMA analysis				
Staining Pattern	Initial Diagnosis	Age of Onset (average years)	Overall Survival (years)	Therapy
**Cell Surface CD44 (+)**				
Cell Surface	AA [1], GBM [14]	43.7±16.9 (5.4–66.3)	1.8±2.1	R,C,S [9];R,S [5];C,S [1]
**Cell Surface CD44 (−)**				
Process	LA [2], AA [2], GBM [14]	57.7±17.1 (17.2–84.8)	2.5±2.5	R,C,S [11];R,S [2];R [1]; S [1]; none [3]
Negative	LA [1], AA [2], GBM [1]	41.2±11.1 (28.8–54.8)	7.8±5.4	R,C,S [3];R,S [1];

[ ]: number of patients; LA: low grade astrocytoma; AA: anaplastic astrocytoma; R: radiation; C: chemotherapy; S: surgery.

\There is no significant difference in the age of onset between the CD44+ and CD44− groups, whereas the overall prognosis for the CD44+ group is significantly poorer.

### Overall CD44 is upregulated in a subset of BTSC in GBM

**Figure 2:**
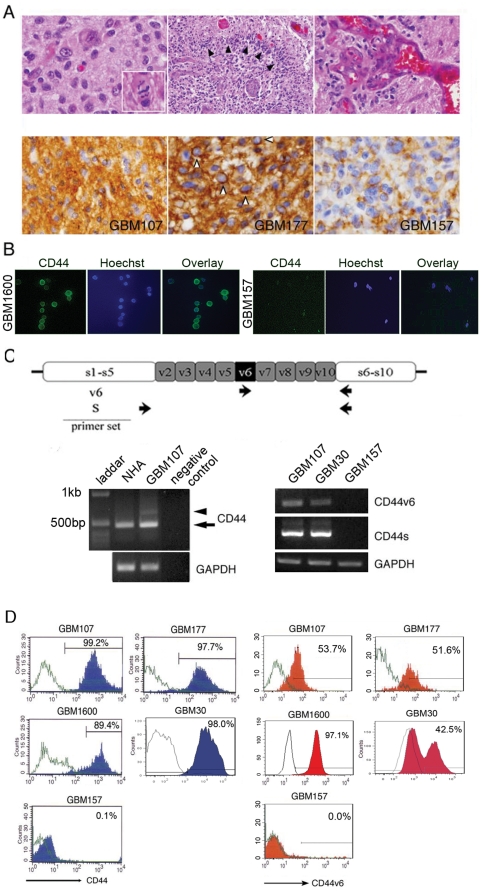
CD44v6 is upregulated in a subset of BTSC in GBM. A: Histopathology of parental tumor of established GBM neurospheres. All GBM samples showed increased cellularity with marked nuclear atypia (upper left panel), magnification ×40, increased mitotic activity (inset), necrosis (upper middle panel),magnification ×20, pseudopalisading (black arrowhead) and vascular proliferation (upper right panel), magnification ×40. GBM107 showed high immunoreactivity with CD44 antibody on process (lower left panel), magnification ×40. GBM177 showed high immunoreactivity with CD44 antibody on cell surface (lower middle panel, white arrowhead) as well as on process. Magnification ×40. GBM157 showed minimal immunoreactivity on process (lower right panel). Magnification ×40. B: GBM 1600 cells revealed CD44 immunostaining (green) (left panel), however, GBM 157 cells were not stained (left panel). Nuclei were counterstained with Hoechst. Magnification ×40. C: A schematic diagram of the CD44 gene with position of primers (upper panel). An expected size of CD44s (arrow) and a longer product (arrowhead) were amplified from GBM107 (lower left panel). GBM107 exhibited CD44s and CD44v6 specific bands (lower right panel). D: Flow cytometry showing the ratio of CD44-expressing cells or CD44v6-expressing cells in each GBM sphere sample.

Recent studies suggest that therapy resistant GBM cells possess tumor stem cell–like properties [1], [33]. We sought to determine a potential role of CD44 in BTSC in GBM. The varied expression levels of pan-CD44 in GBM raised the possibility of the existence of two BTSC subgroups; CD44^high^ and CD44^low^. We established GBM neurosphere cultures from five surgical specimens (Table 2). These specimens met the criteria for GBM; i.e. increased cellularity with marked nuclear atypia (Fig. 2A, upper left panel), increased mitotic activity (Fig. 2A, upper left panel inset), necrosis with pseudopalisading (Fig. 2A, upper middle panel), and vascular proliferation (Fig. 2A, upper right panel). In agreement with the data in a recent study by Anido et al. [14], immunohistochemistry with the pan-CD44 antibody confirmed that four of these tumors (GBM107, 177, 1600, and 30) contained tumor cells with high expression of pan-CD44 (Fig. 2A, lower left and middle panels). Another sample (GBM157) exhibited low expression of pan-CD44 (Fig. 2A, lower right panel).

**Table 2:** Characteristics of GBM samples used for *in vitro* assays.

2. Characteristics of GBM samples for *in vitro* assay				
Patient	Age	Sex	Histology	Location
GBM107	63 Years	Male	GBM	Right Frontal
GBM 1600	34 Years	Male	GBM	Right frontal temporal
GBM 30	65 Years	Male	GBM	Left frontal
GBM 177	47 Years	Male	GBM	Left frontal
GBM 177	54 Years	Female	GBM	Right frontal

We then investigated the expression of pan-CD44 in BTSC derived from GBM. Neurosphere forming capacity under serum-free conditions is a property of BTSC, although cells in neurospheres contain both stem cells and their progeny [4], [5], [33], [34]. When the GBM neurospheres were established from the above 5 samples, both CD44^high^ GBM and CD44^low^ GBM formed neurospheres with no significant differences in their growth rate (data not shown). Immunocytochemical staining revealed that GBM107, 177, 1600 and 30 neurospheres expressed pan-CD44, whereas GBM157 neurospheres were negative for pan-CD44 (Fig. 2B and Figure S1). Thus, these neurospheres samples were found to retain a similar expression pattern of pan-CD44 to the parental tumors.

### CD44 plays a role in the growth of a subset of BTSC

CD44 is known to play a key role in the self-renewal of cancer stem cells in several cancer types including breast cancer, pancreatic cancer, and acute myeloid leukemia [25], [27], [35]. In addition, two recent studies demonstrated the presence of pan-CD44 in GBM and their BTSC as well as its positive role in their growth [14], [36]. Similar to these observations, cell sorting experiments demonstrated that only pan-CD44-positive GBM cells were capable of forming neurospheres in the CD44^high^ GBM samples (GBM107 and 1600) (Figure S 2, left panel), whereas no significant difference in neurosphere-forming potential was exhibited between equal numbers of pan-CD44-positive and -negative cells seeded in serum-free media from the CD44^low^ GBM sample (GBM157) (Figure S2, right panel). Further, treatment of dissociated GBM cells with a monoclonal antibody for pan-CD44 (clone IM7; ABcam), which is widely used to block the pan-CD44 signals *in vitro* [37], [38] resulted in abraded neurosphere formation in the CD44^high^ samples (GBM107 and 1600) (Figure S. 3A, left panel). To exclude non-specific effect caused by incubation with this antibody, we confirmed no significant inhibitory effect on the CD44^low^ sample (GBM157) (Figure S3A, right panel).

To discriminate the effect on either arrest of cell proliferation, induced cell death, and/or differentiation, we analyzed cell growth (Fig. S3B), apoptosis (Fig. S3C) and differentiation (Fig. S3D). Treatment of GBM1600 cells in neurospheres with the CD44-blocking antibody inhibited cell growth (Fig. S3B) without increasing propidium iodide (PI)/AnnexinV positive apoptotic cells (Figure S 3C) or decreasing the proportion of CD133 positive undifferentiated cells (Figure S3D). Taken together, these results suggest that CD44 plays a role in the growth of BTSC in CD44^high^ GBM.

### Presence of CD44v6 in neurospheres derived from CD44^high^ GBM

CD44 has multiple isoforms; the standard form and the variant forms. In human cancer, splice variants of CD44 were frequently identified in advanced stages of tumorigenesis [15], [17], [18]. To determine if any variant forms of CD44 are expressed in BTSC, we performed RT-PCR with a primer set that amplified the standard form (CD44s) and the variant form (CD44v) as different sizes (Fig. 2C, upper panel). GBM neurospheres, but not normal human astrocytes (NHA), yielded only two detectable PCR products, and DNA sequencing demonstrated that the amplified PCR products are CD44s (Fig. 2C, lower left panel, arrow) and CD44v6 (Fig. 2C, lower left panel, arrowhead). Further, by designing a specific primer set to amplify CD44v6 (Fig. 2C, upper panel), we confirmed that neurospheres derived from CD44^high^ GBM, but not from CD44^low^ GBM, expressed CD44v6 (Fig. 2C, lower right panel). We then examined both pan-CD44 and CD44v6 expression in our five GBM neurosphere samples with flow cytometry. All four CD44^high^ GBM neurospheres (GBM107, 177, 1600, and 30) contained a subpopulation of tumor cells expressing CD44v6 (Fig. 2D). In contrast, the CD44^low^ GBM sample (GBM157) exhibited no detectable level of CD44v6-positive cells (Fig. 2D).

### Inhibition of neurosphere formation from CD44^high^ BTSC by targeting CD44v6

**Figure 3:**
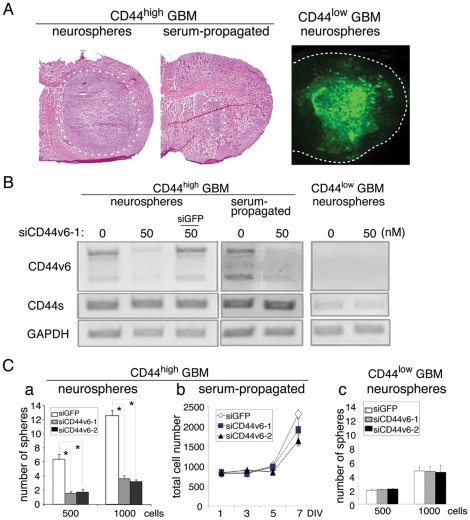
CD44v6 is required for the growth of BTSC in CD44^high^ GBM. A: Brain sections with tumors originated from transplanted GBM neurosphere/serum-propagated cells in mice brains. Neurospheres from CD44^high^ GBM formed remarkable tumor (left panel, surrounded by white dots), while serum-propagated cells formed tiny region (middle panel, surrounded by white dots). Neurospheres from CD44^low^ GBM (GBM157) formed a large tumor (right panel). Magnification ×1. B: Treatment with siRNA decreased the expression of CD44v6 in both neurosphere (left panel) and serum-propagated CD44^high^ GBM (middle panel). C. The numbers of neurospheres (left panel, right panel) or total cells (middle panel) grown after treatment with CD44v6-siRNA. Neurospheres propagated from CD44^high^ GBM neurosphere cells showed statistically significant difference between siRNA and siGFP treated groups (left panel). No statistical difference was seen in growth of serum-propagated cells (middle panel) and neurospheres propagated from CD44^low^ GBM neurosphere cells (right panel). All the experiments were performed in triplicates. *, p<0.05. For neurospheres, Wilcoxon rank test was performed to analyze the data and Bonferroni adjustment was used for pairwise comparison. For serum-propagated cells, Repeated Measurement ANOVA was performed to analyze the trends over time data and Tukey adjustment was used for pairwise comparisons. Results represented as means ± SEM.

To determine the specific role of CD44v6 in CD44^high^ BTSC, we designed two siRNA constructs that target CD44v6, and verified their effects on three different GBM cell samples; neurospheres derived from CD44^high^ GBM1600 and CD44^low^ GBM157, and serum-propagated cells derived from GBM1600. The phenotypic difference between neurospheres and serum-propagated cells was highlighted by their tumorigenic capacity in the xenograft model. When implanted into immunodeficient mice brains, GBM neurospheres from CD44^high^ GBM were capable of forming GBM-like tumors (Fig. 3A, left panel, tumor formation incidence: 3/3). On the other hand, GBM cells from the same tumor that were propagated in serum-containing medium did not possess tumorigenic potential (Fig. 3A, middle panel, tumor formation incidence: 0/3), implying a loss of tumor initiating cells under this condition, in consistence with previous observations [39], [40]. The CD44v6 specific siRNA, when transfected into these two GBM spheres and serum-propagated cells, reduced CD44v6 expression levels, while CD44s expression was not affected in either condition (Fig. 3B, left and middle panels). Knockdown of CD44v6 in CD44^high^ GBM neurospheres resulted in a subsequent reduction in neurosphere formation (Fig. 3C, left panel), whereas growth of the serum-propagated cells was not significantly affected (Fig. 3C, middle panel). As expected, the expression of CD44v6 was undetectable by RT-PCR in the CD44^low^ GBM cells (Fig. 3B, right panel), and siRNA treatment did not affect their neurosphere formation (Fig. 3C, right panel).

### A CD44v6 ligand, osteopontin, activates AKT pathway in a subset of GBM

**Figure 4:**
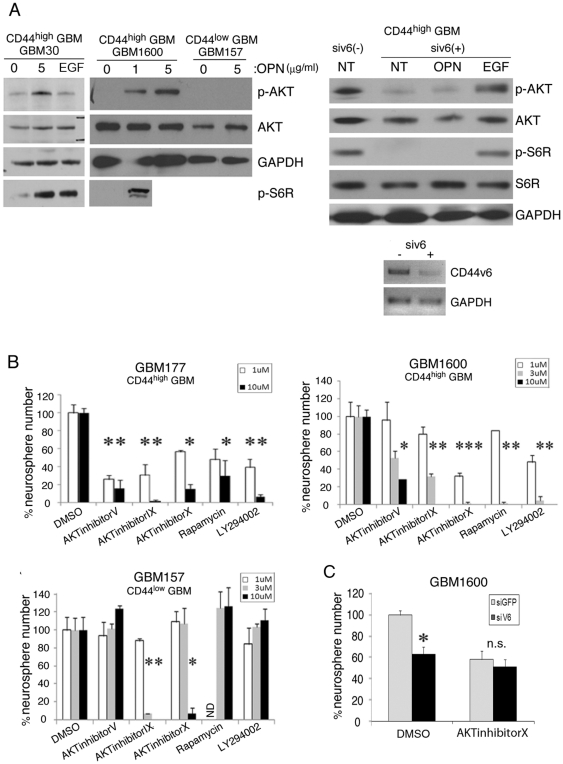
Osteopontin activates AKT pathway in a subset of GBM. A: OPN (1 and 5 µg/ml, 30 minutes) stimulated the AKT pathway in CD44^high^ GBM (GBM1600 and 30) but not in CD44^low^ GBM (GBM157) (upper left panel). In CD44^high^ GBM, S6R was phosphorylated by OPN. GAPDH was used as an internal control. RT-PCR showed treatment with siRNA decreased the expression of CD44v6 mRNA in serum-propagated CD44^high^ GBM (lower right panel). In serum-propagated CD44^high^ GBM cells, AKT and S6R pathway was activated without ligands stimulation (upper right panel). In CD44v6 knocked-down cells, phosphorylation of AKT and S6R was decreased and OPN stimulation (5 µg/ml, 30 minutes) failed to activate both molecules. EGF (10 ng/ml, 15 minutes) caused phosphorylation of AKT and S6R in the absence of CD44v6. “NT” indicates “no ligand treatment”. B: The effect of various AKT inhibitors on neurosphere formation derived from CD44^high^ GBM (upper left panel, GBM177 and upper right panel, GBM1600) and CD44^low^ GBM (lower left panel, GBM157). “ND” in (lower left panel) indicates “not determined”. C: Neurosphere numbers were significantly decreased with siRNA for CD44v6 in DMSO-treated, but not in AKT inhibitor X-treated GBM 1600 cells00. All the experiments were performed in triplicates. “n.s” indicates “not significant”. *, p<0.05. Two sample t-test with bonferroni adjustment was performed to compare the groups. Results represented as means ± SEM.

To further gain insight into their signaling pathway, we sought to identify their downstream targets and to characterize the distinct mechanism of CD44v6 in BTSC growth. OPN is a ligand for CD44v6, and, in some hematopoietic malignancies, activation of CD44v6 by OPN contributes to tumor cell survival via the PI3K/AKT pathway [41]. Therefore, we investigated whether OPN triggers AKT activity in neurospheres derived from GBM30 and GBM1600 (both CD44^high^), as well as GBM157 (CD44^low^) (Fig. 4A, upper left panel). Incubation with OPN resulted in an appreciable increase in phosphorylated AKT in spheres derived from both GBM30 and GBM1600 spheres but not in the GBM157 spheres (Fig. 4A, upper left panel). As expected, OPN treatment also activated S6R, a downstream target of AKT, in both GBM30 and GBM1600 spheres (Fig. 4A, upper left panel). To elucidate the signaling pathways in CD44v6-expressing GBM cells, we knocked down CD44v6 with siRNA in GBM1600 cells (Fig. 4A, lower right panel). Untreated cells showed activation of the AKT and S6R pathway without ligand stimulation (Fig. 4A, upper right panel). With CD44v6 knockdown, both phosphorylated AKT and S6R were under detectable level and OPN failed to activate AKT and S6R, while EGF substantially increased both molecules (Fig. 4A, upper right panel). These observations prompted the question of whether sensitivity to the PI3K/AKT inhibitors is different between CD44v6-expressing BTSC and CD44v6-negativeBTSC. To address this question, we performed neurosphere-forming assay using 3 GBM samples (Fig. 4B). For inhibition of the PI3K/AKT pathway, we used five different small molecules. In both GBM177 cells and GBM1600 cells, a dose-dependent inhibition for neurosphere formation was observed for all five inhibitors (Fig. 4B, upper left and right panels). In contrast, GBM157 cells were relatively resistant to the treatment (Fig. 4B, lower left panel).

We also addressed an assumption that if AKT is the major downstream target of CD44v6, the phenotype of CD44v6 knockdown in GBM cells should be masked by inhibition of AKT. Therefore, we combined transfection of siRNA for CD44v6 and AKT inhibitor X treatment in GBM 1600 cells (Fig. 4C). AKT inhibitor X-treated GBM1600 cells did not yield significant difference in the number of neurospheres with or without knockdown of CD44v6. These data suggest that a subset of BTSC in GBM may depend on CD44v6 and the action of CD44v6 is mediated through AKT.

### CD44v6 in normal stem cells and brain tumor stem cells in the murine model

**Figure 5:**
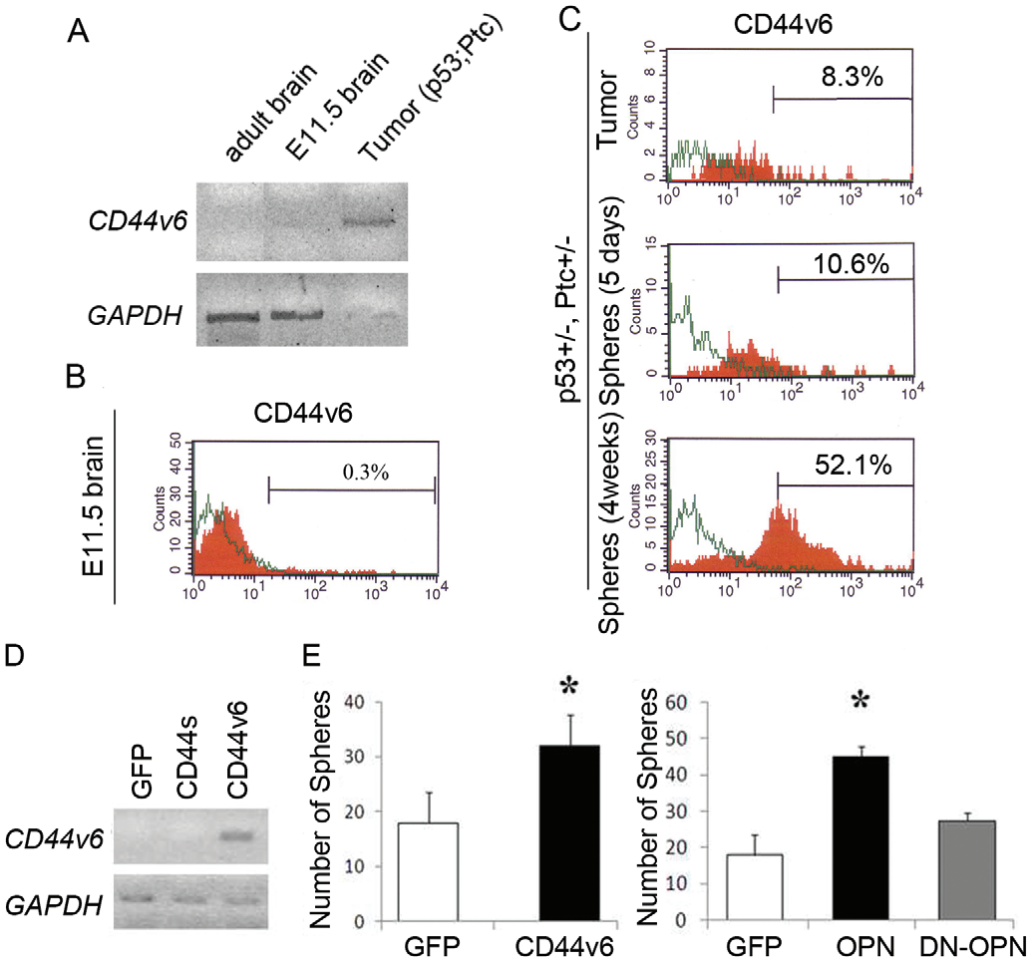
The OPN-CD44v6 axis plays a positive role in growth of stem-like tumor cells in p53/Ptc double heterozygous mice. A: RT-PCR detection of CD44v6 in indicated samples. B: Flow cytometry using CD44v6 antibody with E11 cortical progenitors. C: Flow cytometry using CD44v6 antibody with tumor cells in p53/Ptc double heterozygous mouse brains. D: Bands represent CD44v6, as detected by RT-PCR. E: Graphs indicate the effect of overexpression of CD44v6 or OPN on tumor neurosphere formation. All the experiments were performed in triplicates. *, P<0.05, ANOVA followed by post-hoc t test. Results represented as means +/− SEM. Abbreviations: DN-OPN: Dominant negative form of OPN, ptc: sonic hedgehog receptor patched, E11.5: Gestation age of 11.5.

Recent studies have suggested that overlapping genes andsignaling pathways regulate the proliferation of both tumor stem cells and normal stem cells [42], [43]. Targeting pathways that are essential for proliferation of both tumor and normal stem cells may result in the same outcome for both. To determine whether CD44v6 might be a potential target in BTSC without affecting the maintenance of proliferating normal stem cells, we used a mouse model of brain tumor. Mice heterozygous for a mutation in the gene encoding the sonic hedgehog receptor patched 1 (Ptc), known to form spontaneous intracranial malignant tumors, were bred with mice heterozygous for a tumor suppressor gene, p53 (Ptc+/−, p53+/−). Diamandis et al. [44] have demonstrated that neurospheres derived from these mouse tumors are enriched with multipotent self-renewing brain tumor stem cells. We, therefore, used these mice to further characterize CD44v6 in BTSC and their normal counterpart. To enrich for normal neural stem cells, we cultured neurospheres from the subventricular zone cells using the wild type mice at the gestation age of 11.5 (E11.5). RT-PCR detected mouse CD44v6 in tumor neurospheres, but not in normal mouse brain samples (Fig. 5A). Flow cytometry analysis exhibited similar results (Fig. 5B and C). Although neurospheres derived from the E11.5 cortices had 14.9% of the cells labeled with the pan-CD44 antibody, these normal progenitors did not express appreciable CD44v6 (0.3% compared to the negative control samples) (Fig. 5B). In contrast, tumor specimens in the p53+/−, Ptc+/− mice had a much greater fraction of the CD44v6-expressing cells (8.3%) (Fig. 5C, upper panel). Interestingly, when these tumor cells were cultured in serum-free medium to form neurospheres, the fraction of CD44v6-positive cells increased over the period of one month (8.3% vs. 52.1%), suggesting the preferential proliferation of CD44v6-positive cells in conditions that enriched for BTSC (Fig. 5C middle and lower panels). This result was consistent with the increased expression of stem cell-associated proteins, including Nestin and Sox2 in these cultures (data not shown). We then investigated the function of CD44v6 and its ligand, OPN, in these mouse tumor spheres (Fig. 5D and E). Both overexpressions of CD44v6 and OPN, but not the dominant negative form of OPN, significantly increased sphere formation derived from the tumor cells (Fig. 5E). These data suggests that the OPN-CD44v6 axis plays a positive role in proliferation of BTSC in a subset of GBM cases.

## Discussion

An accumulating body of evidence suggests that tumor heterogeneity exists in various types of cancers, including GBM [4], [6], [12]. However, differential regulatory molecules and pathways specific to each tumor type are poorly understood. In agreement with a recent study by Anido et al. [14], we identified a subpopulation of GBM in which CD44 expression was upregulated (CD44^high^ GBM). CD44^high^ GBM demonstrated correlation with poorer clinical prognosis. Xu et al. [13] showed combined treatment of mouse intracranial tumors derived from a glioma cell line with CD44 antagonist and the current first line chemotherapy, temozolomide, prolonged survival of mice. Temozolomide is known to preferentially kill non-stem GBM cells [14]. Collectively, these data raise a possibility that CD44-expressing GBM cells are relatively therapy resistant and likely a reasonable therapeutic target, especially in recurrent GBM tumors that survived over the current therapies. However, the case number of our comparison is still limited and a definite conclusion should be drawn with more accumulated data set in the future.

Here, we provide the first evidence for the presence of CD44v6 in BTSC derived from CD44^high^ GBM. Khan et al. [20] suggested that CD44v6 regulates the aggressiveness of breast cancer cells. We found that both CD44v6 overexpression and OPN overexpression increased sphere forming ability of mouse intracranial tumor cells. In turn, knockdown of CD44v6 resulted in reduced growth of human BTSC derived from CD44^high^ GBM but not from CD44^low^ GBM *in vitro*. On the other hand, the effect on serum-propagated cells from the matched CD44^high^ GBM was less prominent and not statistically significant. However, these data need to be carefully interpreted, as serum-propagated human GBM cells do express CD44v6 (Fig. 5B). It is possible that targeting CD44v6 may reduce the growth of both BTSC and non-stem GBM cells with different potency. Future study is needed to address this question.

Interestingly, CD44v6 was not detected in normal mouse brains or neural progenitors (Fig. 5A and B). The clear difference of CD44v6 expression between normal neural cells and glioma cells may indicate a potential therapeutic target molecule in GBM. The data in this study suggest that, in a subset of GBM, CD44v6 may preferentially target BTSC in GBM and such a treatment may not significantly affect the normal cells in the brain.

Several studies have demonstrated that elevated AKT expression in GBM correlates with poor clinical prognosis [45], [46], [47]. Recently, Gallia et al. [48] exhibited some data suggesting that inhibition of the AKT pathway eliminates the growth of GBM and GBM stem-like cells, implicating a role for AKT in BTSC survival and proliferation. Additionally, Eyler et al. [49] provided evidence that treatment of BTSC with AKT inhibitors induces apoptosis, decreases motility and invasiveness of BTSC *in vitro*, and inhibits tumor growth *in vivo* in a xenograft model. In colon cancers, action of CD44v6 is likely mediated through the AKT pathway [17]. Consistent with these findings, our data suggest that downstream targets of the CD44v6 action in BTSC include the AKT-mediated signaling pathway (Fig. 4A). Knockdown of CD44v6 eliminated *in vitro* growth of BTSC in CD44^high^ GBM (Fig. 3C). In addition, a ligand for CD44v6, OPN, phosphorylated AKT in these cells (Fig. 4A). These data may indicate that the activity of the AKT-mediated pathway may, at least in part, depend on the OPN-CD44v6 status. The experiments using PI3K/AKT inhibitors exhibited that various inhibition of AKT affected the neurosphere formation in CD44^high^ GBM cells, while CD44^low^ GBM cells appeared to be relatively less dependent on the AKT pathway (Fig. 4B). Collectively, these results prompted a speculation that CD44v6-mediated AKT pathway plays a role in proliferation, specifically in CD44^high^ BTSC.

Another question still remains open. Both CD44^high^ and CD44^low^ GBM cells formed neurospheres without significant difference in their growth rate. Neurospheres derived from GBM157 (CD44^low^) had similar tumorigenic potential in comparison to CD44^high^ neurosphere samples. These data suggest that CD44 and CD44v6 are not universally expressed by sphere-forming tumorigenic stem-like GBM cells. To determine what extent of GBMs are dependent on the CD44v6/AKT pathway and the mechanisms underlying the interaction between CD44v6 and AKT, future studies with larger numbers of GBM specimens and other ligands of CD44v6 will be required.

In conclusion, we identified that CD44^high^ GBM relied on their variant form 6 for proliferation and conferred a shorter survival period on the patients. Our data suggested that the mechanism of the CD44v6 action on BTSC proliferation is mediated, at least in part, through its interactions with OPN and the subsequent activation of the AKT pathway. Collectively, targeting the CD44v6 pathway through inhibition of CD44v6 itself or its ligands appears to be a promising strategy for future therapeutic development for patients with CD44^high^ GBM.

## Supporting Information

Figure S1 **CD44 is expressed by a subset of patient-derived GBM sphere samples.** Immunocytochemistry indicates CD44 signals (green) in GBM samples. Hoechst is used for nuclear staining.
(TIF)

Figure S2 **CD44-expressing GBM cells have higher sphere-forming ability in a subset of GBM samples.** CD44-positive cells sorted from CD44^high^ GBM sphere showed statistically significant increase of sphere formation than CD44-negative cells (lower left panel). Cells from CD44^low^ GBM sphere showed no statistical difference (lower right panel). All the experiments were performed in triplicates. *, p<0.05, one way analysis of variance followed by post-hoc *t* test. Results represented as means ± SEM.
(TIF)

Figure S3 **CD44 plays a key role in the growth of a subset of BTSC.** A: Inhibition of CD44 by anti-CD44 neutralizing antibody. Neutralized GBM sphere cells from CD44^high^ GBM decreased the sphere formation (upper left panel). Cells from CD44^low^ GBM showed no difference (upper right panel). B: Neutralized cells from CD44^high^ GBM decreased the cell growth. C, D: Neutralized cells from CD44^high^ GBM did not show the shift of Propidium Iodide (PI)/AnnexinV staining pattern (C) and CD133-positive undifferentiated cell ratio (D). All the experiments were performed in triplicates. *, p<0.05, one way analysis of variance followed by post-hoc *t* test. Results represented as means ± SEM.
(TIF)
