# Supplementary figures and images for: CD44v6 Regulates Growth of Brain Tumor Stem Cells Partially through the AKT-Mediated Pathway
Source: PLoS One. 2011 Sep 6;6(9):e24217. doi: 10.1371/journal.pone.0024217 (PMC3167830; doi:10.1371/journal.pone.0024217)

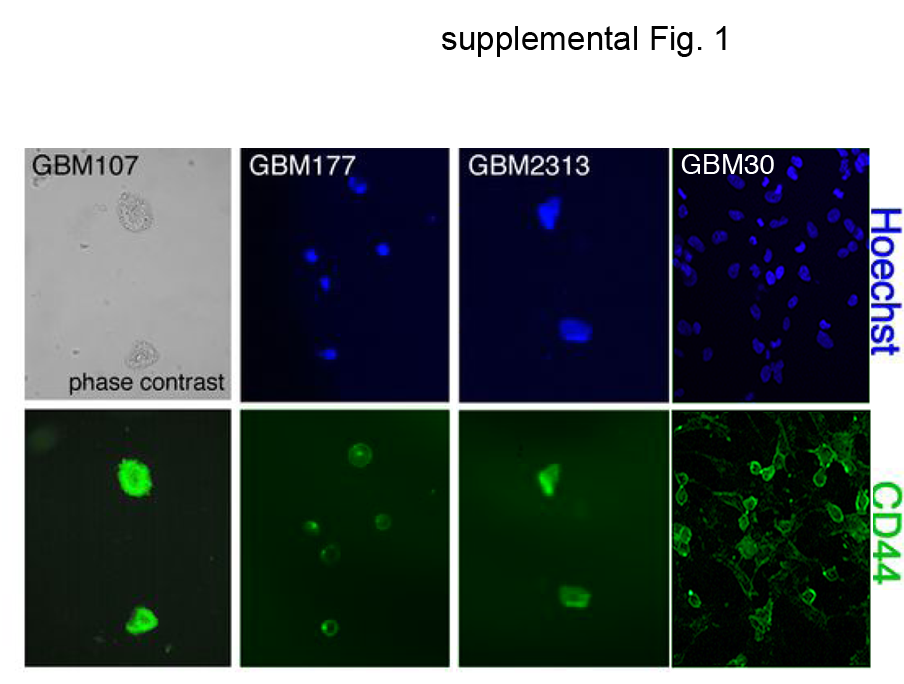

Supplement: Figure S1 — CD44 is expressed by a subset of patient-derived GBM sphere samples. Immunocytochemistry indicates CD44 signals (green) in GBM samples. Hoechst is used for nuclear staining. (TIF) [file pone.0024217.s001.tif]

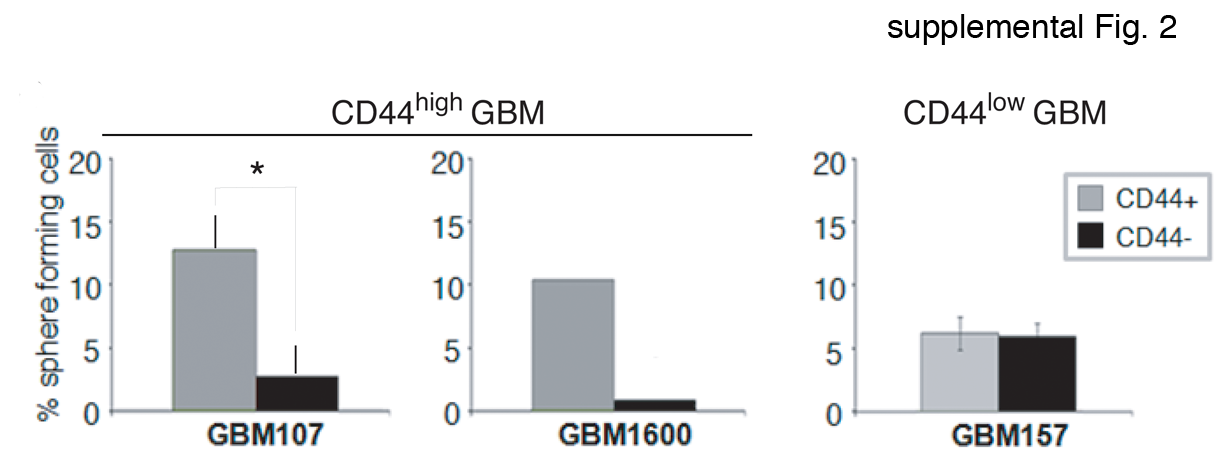

Supplement: Figure S2 — CD44-expressing GBM cells have higher sphere-forming ability in a subset of GBM samples. CD44-positive cells sorted from CD44high GBM sphere showed statistically significant increase of sphere formation than CD44-negative cells (lower left panel). Cells from CD44low GBM sphere showed no statistical difference (lower right panel). All the experiments were performed in triplicates. *, p<0.05, one way analysis of variance followed by post-hoc t test. Results represented as means ± SEM. (TIF) [file pone.0024217.s002.tif]

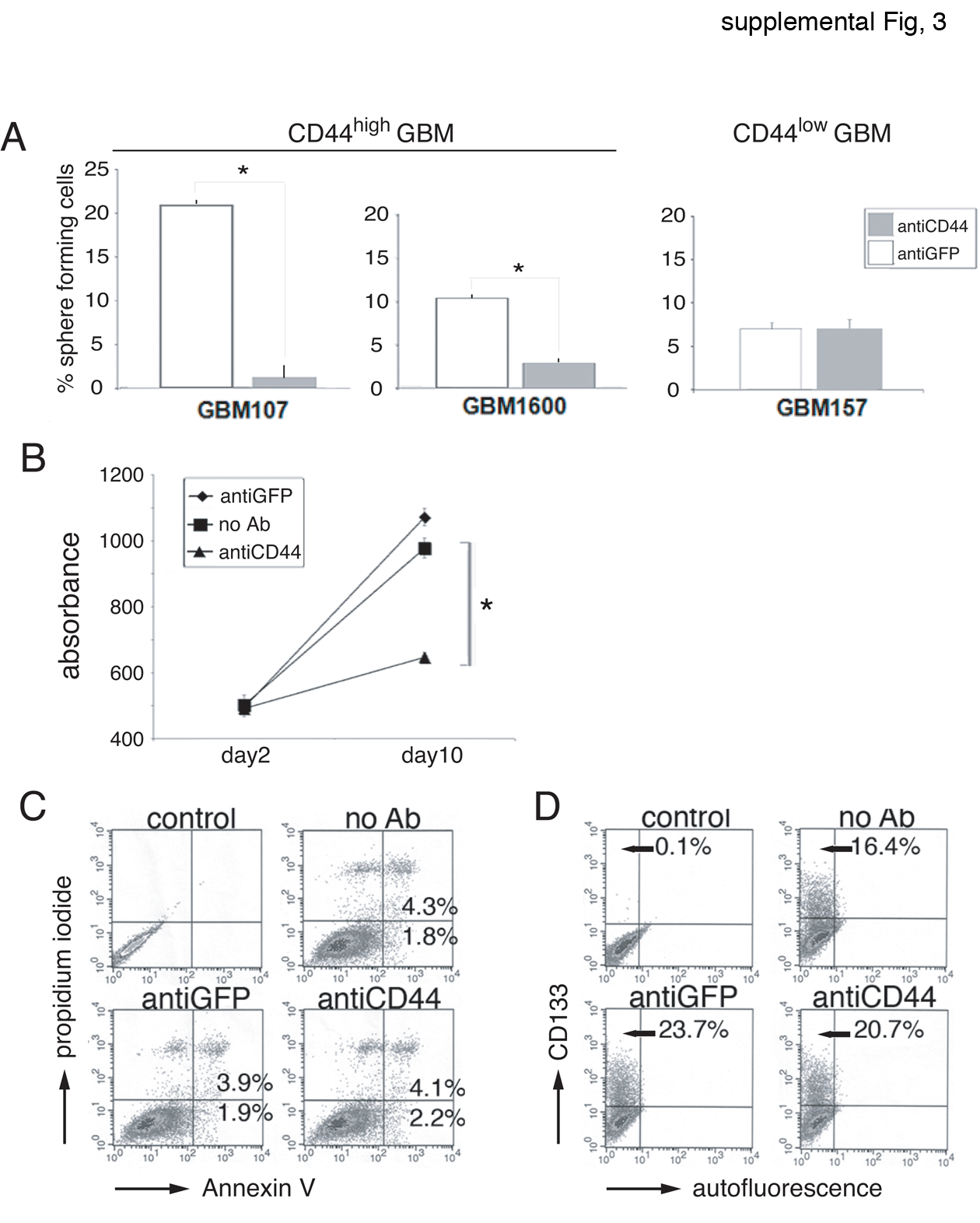

Supplement: Figure S3 — CD44 plays a key role in the growth of a subset of BTSC. A: Inhibition of CD44 by anti-CD44 neutralizing antibody. Neutralized GBM sphere cells from CD44high GBM decreased the sphere formation (upper left panel). Cells from CD44low GBM showed no difference (upper right panel). B: Neutralized cells from CD44high GBM decreased the cell growth. C, D: Neutralized cells from CD44high GBM did not show the shift of Propidium Iodide (PI)/AnnexinV staining pattern (C) and CD133-positive undifferentiated cell ratio (D). All the experiments were performed in triplicates. *, p<0.05, one way analysis of variance followed by post-hoc t test. Results represented as means ± SEM. (TIF) [file pone.0024217.s003.tif]
